# The Path to Eliminating Cervical Cancer in Canada: Past, Present and Future Directions

**DOI:** 10.3390/curroncol29020095

**Published:** 2022-02-14

**Authors:** Hannah Caird, Jonathan Simkin, Laurie Smith, Dirk Van Niekerk, Gina Ogilvie

**Affiliations:** 1 BC Centre for Disease Control, Vancouver, BC V5Z 4R4, Canada; hannah.caird@bccdc.ca; 2 BC Cancer, Vancouver, BC V5Z 4E6, Canada; jonathan.simkin@bccancer.bc.ca (J.S.); laurie.smith@bccancer.bc.ca (L.S.); dvanniek@bccancer.bc.ca (D.V.N.); 3 Faculty of Medicine, School of Population and Public Health, University of British Columbia, Vancouver, BC V6T 1Z3, Canada; 4 Women’s Health Research Institute, Vancouver, BC V6H 2N9, Canada

**Keywords:** cervical cancer, cancer incidence, HPV, cancer control and prevention, elimination

## Abstract

Cervical cancer remains a common cancer affecting women in Canada. While cervical cancer incidence and mortality in Canada have declined for several decades due to the success of organized, provincial cervical cancer screening programs, further decreases will require enhancement of primary, secondary, and tertiary prevention efforts. The present commentary provides a historical overview of cervical cancer trends in Canada, presents current statistics on cervical cancer incidence, mortality and survival, and discusses future directions in relation to cervical cancer elimination.

## 1. Introduction

While cervical cancer incidence in Canada has declined for several decades due to the success of organized cervical cancer screening, cervical cancer continues to impact the lives of women, and all people with a cervix, across Canada [1]. Women under 50, who have critical roles in the family, society, and the economy, account for over half of new cervical cancer cases in Canada [1]. When these women are incapacitated as a result of cervical cancer, the consequences are wide-reaching and significant. In 2018, women’s earnings accounted for 46.7% of the family income, an increase of 22.2% since 1976 [2]. As well, the proportion of women who earn more than half of their family income is 40.9%, up from 17.4% in 1976 [2]. Loss of this family income when a woman is sick or dies is just one of the substantial, irreversible consequences of cervical cancer on children and families, and as such, preventing cervical cancer has broad societal implications.

Following the call to action in 2018, in 2020, the World Health Organization launched a global strategy to accelerate the elimination of cervical cancer. Specifically, the goals call for improved early detection, diagnosis, and treatment, prioritizing vaccination, and expanded research [3]. The Canadian Partnership against Cancer also developed an action plan to eliminate cervical cancer in Canada by 2040 with strategies that include the improvement of HPV vaccination rates, the implementation of HPV primary screening, and enhanced efforts for follow-up of abnormal results [4]. The purpose of this commentary is to provide a historical overview of cervical cancer trends in Canada, its impact on women, current statistics on cervical cancer incidence, mortality and survival, and discuss future directions in relation to cervical cancer elimination.

## 2. Trends in Incidence, Mortality, Stage of Diagnosis and Survival

Age-standardized incidence rates (ASIRs) were obtained from Statistics Canada Table 13-10-0747-01, which includes data from the Canadian Cancer Registry [5]. Data reflect cervical cancer ASIRs from 1992 to 2018 for Canada excluding Quebec. Data from Quebec is unavailable for significant periods during the overall period and, therefore, were excluded. The 2011 Canadian Census age structure was used as the population standard. Data were available from 1992 to 2018.

Age-standardized mortality rates (ASMRs) were calculated using direct age-standardization from mortality counts and population estimates data. Cervical cancer mortality counts among all Canadians were obtained from Statistics Canada Table 13-10-0392-01, which includes data from the Canadian Vital Statistics Death Database [6]. Population estimates were obtained from Statistics Canada Table 17,100,005 [7]. For this, 19 five-year age groups were used (0–4, 5–9, …, 90+ years) and the 2011 Canadian Census age structure was used as the population standard. The earliest year of available data was 2000.

## 3. Incidence

**Figure 1:**
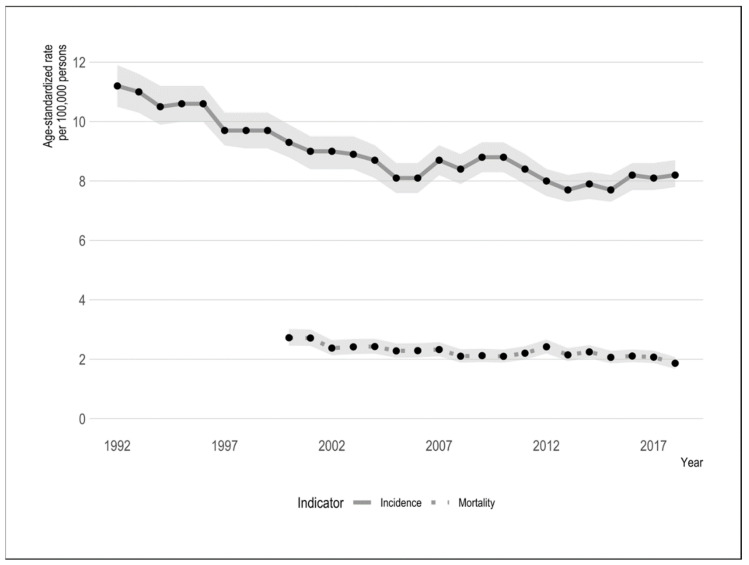
Canadian cervical cancer age-standardized incidence and mortality rates, 1992–2018. Grey ribbons reflect 95% confidence intervals.

Cervical cancer incidence has steadily declined since the 1970s, with annual percentage changes (APC) ranging from −8.8% to −0.33% [8]. In 1984, the age-standardized incidence rate (ASIR) of cervical cancer was 13 per 100,000 and has declined to an estimated 7.1 per 100,000 persons in 2020 [8]. Nationally, ASIR trends reported for 1984–2015 showed an average annual percentage change (AAPC) of −1.8% (95% confidence intervals (CI) = −1.3 to −2.3) [9]. Despite a sharp decline in the trend early on, the downward trend has slowed in recent periods—Brenner et al. reported a decline from 9.0 per 100,000 persons in 2002 to 8.0 per 100,000 persons in 2016 [9]. Incidence trends have tended to fluctuate slightly over this period. These trends are illustrated in Figure 1.

Similar trends have been observed internationally as well—in the United States, incidence rates declined steadily from 1975–1982 from 14.8 to 10.6 per 100,000, remained stable through 1990 and declined until 2006, after which rates remained stable at around 6.8 per 100,000 [10]. Cervical cancer incidence rates in the United Kingdom decreased by 25% between 1993–1995 and 2015–2017, from 14 to 9 per 100,000 [11]. In Australia, from 1982–2016, cervical cancer incidence decreased from 14.3 cases per 100,000 to 7.1 cases per 100,000 [12].

It is now well established that a persistent infection with an oncogenic genotype of the human papillomavirus (HPV) is necessary for the development of cervical cancer and there are substantial prevention opportunities with the availability of HPV vaccines [13]. As in other regions globally, HPV 16 and 18 are the most common types of HPV in Canada, causing roughly 70% of cervical cancers [14]. An additional 20% of cervical cancers are caused by HPV types 31, 33, 35, 45, 52 and 58 [14]. Among Canadian women with normal cytology, HPV 16/18 prevalence was 6.2%, while among women with low-grade (CIN1) and high-grade (CIN2+) lesions, HPV 16/18 prevalence was 33.5% and 67%, respectively [14]. In Canada, cervical pre-cancer (CIN2 or CIN3) is often detected through screening and treated prior to progression to cervical cancer. As a result, one of the earliest surrogates for effectiveness of HPV vaccination is assessment of CIN2 or CIN3 (CIN2+) rates in vaccinated women. Preliminary research has found that women who received a complete HPV vaccination schedule between the ages of 9 to 14 had a lower incidence of CIN2+ compared to those who were not vaccinated, over a 7-year follow-up period [15]. A recent population-based cohort study out of Sweden showed an incidence rate ratio of 0.12 (95% CI: 0.00–0.34) of invasive cervical cancer in women vaccinated before the age of 17 compared to unvaccinated women, and 0.47 (95% CI: 0.27–0.75) among women vaccinated between the ages of 17 and 30 [16]. This substantial decrease in risk of pre-cancerous lesions of the cervix in women vaccinated with a quadrivalent HPV vaccine shows promise for other countries, including Canada, with comprehensive school-based immunization programs. As of 2018/19, all Canadian jurisdictions other than Quebec offer nonavalent HPV vaccines to both boys and girls, offering additional protection compared to the quadrivalent vaccine [17]. Prior cohorts who received the quadrivalent vaccine are expected to be well-protected as it protects against HPV 16/18, which is known to cause 70% of cervical cancers [14].

## 4. Mortality

The age-standardized mortality rate (ASMR) for cervical cancer was estimated to be 2.0 per 100,000 persons in 2019 [8]. Overall, the ASMR has declined by an average of 2.1% since 1984 however, stronger declines were observed in earlier periods [1]. Cervical cancer ASMRs decreased by 2.8% per year from 1984 to 2008, and then stabilized between 2008–2015 (average annual percent change = 0.2) [1]. Overall, trends in mortality have declined significantly over a long period of time and are likely reflective of early detection of cancer and advances in treatment.

There were a total of 2026 deaths related to cervical cancer between 2015 and 2019, averaging 405 deaths per year [6]. However, the largest number of total deaths occur for women aged 50–54 years, at 226 total deaths from 2013–2017 [6]. Women between 50–54 accounted for 21.3% of cervical cancer deaths during this time [6].

## 5. Incidence by Age

Cervical cancer was the third most common cancer among women aged 25–34 years, accounting for 10.1% of all cancers [18]. It remains the third most common cancer among women aged 35–44 years, accounting for 6.6% of new cancers [18]. The median age at diagnosis of cervical cancer is 47 years [18].

A total of 5710 women in Canada (excluding Quebec) were diagnosed with cervical cancer from 2014–2018, at an average of 1142 cases per year [18]. Women between the ages of 25 and 69 years accounted for 87.2% of all new cervical cancer cases between 2014–2018 [18]. Trends in ASIRs were reported to differ by age in Canada, where there have been downward trends across all age groups other than those aged 30–39 [8].

## 6. Stage of Diagnosis and Survival

Presently in Canada, cervical cancer is more likely to be diagnosed at an early stage, reflective of the positive impacts of organized cervical cancer screening programs. Overall, 54.4% of cervical cancers are diagnosed at stage I, 13.4% at stage II, 16.5% at stage III and 11.8% at stage IV [19]. The distribution of stage of diagnosis differs by age. Generally, the percentage of cases diagnosed at stage I decreases with age while the proportion of stage IV cases increases with age. For women older than 70 years, 23% of cervical cancers are diagnosed at stage IV [19].

The 5-year net survival, or probability of survival for the specific outcome of cervical cancer, is 72% in Canada [1]. This is higher than international peer countries such as the United States (66.3%), and the United Kingdom (61.4%) [10,11]. Between 1992–1994 and 2012–2014, an improvement in the age-standardized 5-year net survival was observed in Canada (2.4%) [1].

## 7. Screening and Social Disparities

Despite the existence of publicly funded cervical cancer screening programs across Canada, inequities in access to screening services persist. Decreases in incidence rates are related to the success of organized, provincial cervical cancer screening programs in most provinces [1]. All provinces except for the Yukon, Northwest Territories, Nunavut and Quebec have an organized cervical cancer screening program [15]. HPV vaccination through publicly funded school-based programs have now been in existence for over a decade [20]. When the HPV vaccine is offered to school-age children, information about HPV and its relationship to cervical cancer is sent home to parents with the immunization consent form [21]. Adherence to recommended cervical cancer screening guidelines varies by province, ranging from 63% in Saskatchewan to a high of 71% in Newfoundland and Labrador, over a 42-month period [19]. After correcting for hysterectomy, participation was highest for those aged 40–49 (77.2%) and lowest for those aged 60–69 (63.7%) [19].

Of those diagnosed with invasive cervical cancer in Canada from 2011–2013, 37% had last undergone pap screening more than 5 years ago, or never, suggesting the need to reach under-screened populations [19]. Under-screened groups in Canada include those with lower educational attainment, lower socioeconomic status, those who are foreign-born, and those of Indigenous identity [22]. These same disparities were reported in the actual occurrence of invasive cervical cancer, where visible minority, Indigenous, unmarried, and rural women have higher-than-expected rates of cervical cancer [23]. Despite the availability of publicly funded programs, social disparities in equitable screening access and cancer incidence persist, suggesting that there continue to be socioeconomic and sociocultural barriers in cervical cancer prevention and control programming [23]. Strategies to reach under-screened groups and eliminate barriers to screening must be implemented, such as mobile screening units, mail-in self-collection HPV sampling kits, community health workers who provide education and information, media campaigns, and direct telephone calls [24,25]. As well, closer monitoring of groups that are known to be perpetually under-screened, including Indigenous and visible minority populations, should be considered in order to ensure they are accessing screening at the recommended schedule [26].

## 8. Future Directions towards Cervical Cancer Elimination

The WHO set an ambitious “90/70/90” target to be achieved globally by 2030 in order to be on track for cervical cancer elimination by 2040: 90% of girls vaccinated against HPV by age 15, 70% of women screened using high-precision testing by age 35 and 45, and 90% of cervical cancers identified and treated [3]. The Canadian Partnership Against Cancer hosted a pan-Canadian summit on the elimination of cervical cancer with local experts and a Canadian-specific target was set to 90/90/90 following a multi-year consultation process with specialty committees [4]. These targets are similar to WHO goals, but with Canada aiming for 90% of eligible women screened with an HPV test and up-to-date on their cervical cancer screening [4].

For Canada to achieve these goals, a combination of strategies must be considered that improve primary, secondary, and tertiary prevention. In terms of primary prevention, efforts are required to increase uptake of HPV vaccination. Expansion of the HPV vaccine to broader cohorts, with population-based monitoring of vaccine uptake can help accelerate elimination. Enhanced efforts are required to better understand and address vaccine hesitancy [27]. Hesitation and misinformation specific to HPV vaccinations should be combatted through public information campaigns and advice from physicians. Secondary prevention strategies include the transition from a cytology-based approach to HPV testing as the primary modality in cervix screening. Research has demonstrated that HPV testing has superior sensitivity for detecting CIN2+ disease compared to cytology testing and leads to lower rates of CIN2+ in future years [28]. HPV testing also provides the opportunity to offer alternative approaches to clinician-based screening through self-collection methods [25]. The underlying reasons for not accessing cervix screening, such as barriers to healthcare access and lack of awareness of the reasons for or benefits of screening, among others, must be specifically addressed. Efforts to eliminate cervical cancer must enhance outreach to under-served populations in order to ensure the benefits of screening programs are available and accessible to all women who require screening. HPV self-sampling can be used as a strategy to increase screening participation, and has been shown to consistently improve compliance in never-screened and under-screened women [29]. Regardless of the screening technology used, screening uptake rates need to be increased to ensure the under-screened, those at most risk for cervical cancer, have access to and receive cervix screening.

Elimination of cervical cancer will not be reached among all groups in the population if existing inequities are not addressed. More work is needed to better understand and address current sociodemographic differences in cervical cancer screening participation, incidence, treatment and mortality.

## 9. Conclusions

Cervical cancer incidence in Canada has declined overall; however, the downward trend has slowed. Further decreases in incidence require improving HPV vaccine uptake, incorporating novel HPV-based screening technologies, increasing participation in cervical cancer screening and addressing inequities in access to and availability of screening services between population groups.

## Data Availability

No new data were created or analyzed in this study. Data sharing is not applicable to this article.
